# A proposed mechanism for the MDMA-mediated extinction of traumatic memories in PTSD patients treated with MDMA-assisted therapy

**DOI:** 10.3389/fpsyt.2022.991753

**Published:** 2022-10-12

**Authors:** Robert J. Sottile, Thomas Vida

**Affiliations:** Department of Medical Education, Kirk Kerkorian School of Medicine at UNLV, University of Nevada Las Vegas, Las Vegas, NV, United States

**Keywords:** MDMA, PTSD, psychedelic-assisted psychotherapy, fear extinction, fear anxiety and relief

## Abstract

Post-traumatic stress disorder (PTSD) is a devastating psychiatric disorder afflicting millions of people around the world. Characterized by severe anxiety, intrusive thoughts, pervasive nightmares, an assortment of somatic symptoms, associations with severe long-term health problems, and an elevated risk of suicide, as much as 40–70% of patients suffer from refractory disease. 3,4-Methylenedioxy-methamphetamine (MDMA), like classic psychedelics such as psilocybin, have been used to enhance the efficacy of psychotherapy almost since their discovery, but due to their perceived potential for abuse and inclusion on USFDA (United States Food and Drug Administration) schedule 1, research into the mechanism by which they produce improvements in PTSD symptomology has been limited. Nevertheless, several compelling rationales have been explored, with the pro-social effects of MDMA thought to enhance therapeutic alliance and thus facilitate therapist-assisted trauma processing. This may be insufficient to fully explain the efficacy of MDMA in the treatment of psychiatric illness. Molecular mechanisms such as the MDMA mediated increase of brain-derived neurotrophic factor (BDNF) availability in the fear memory learning pathways combined with MDMA's pro-social effects may provide a more nuanced explanation for the therapeutic actions of MDMA.

## Introduction

3,4-Methylenedioxy-methamphetamine (MDMA), much like classical psychedelics such as psilocybin, has recently received significant attention as a possible novel therapeutic for treatment-resistant psychiatric illnesses such as PTSD (1, 2). The (classical) psychedelics including psilocybin, N,N′-dimethyltryptamine (DMT), and lysergic acid diethylamide (LSD), have been defined as molecules that produce an altered state of consciousness, primarily mediated by activation of the serotonergic 5HT_2A_ receptor (3, 4). MDMA, while also a hallucinogenic, primarily acts through the inhibition of monoamine reuptake and is not considered a classical psychedelic but rather an “entactogen” due to its powerful pro-social effects (3, 5). MDMA, the primary psychoactive compound in the street drug Ecstasy (or Molly), remains a schedule I medication indicating high abuse potential without an approved medical use despite FDA approval as a breakthrough therapy for PTSD in 2017 (6, 7). Designation as a breakthrough therapy, however, does not guarantee that a pharmaceutical will be approved for use, and only indicates that preliminary clinical evidence suggests that the drug may have a substantial benefit in the improvement of a primary endpoint over current therapies in the treatment of a life-threatening disease (6, 8). Substance misuse in the United States is a significant problem, with an estimated annual financial impact of at least $420 billion dollars, $120 billion of the total in healthcare related expenses (9). MDMA can indeed be a drug of abuse, with frequent users developing tolerance and withdrawal symptoms after sudden cessation of use (10). Further, the consequences of MDMA use are complicated by poly-substance abuse and the frequent impurity of street Ecstasy (10–12).

Thus, despite growing evidence that MDMA may have a role in treating serious, debilitating mental illness, the drug is still primarily associated with raves, intoxication, and abuse (13, 14). This may be similar to the stigma associated with the treatment of ADHD with dextroamphetamine despite 40 years of evidence supporting the efficacy of stimulant therapy for the disorder (15, 16). It is therefore of some importance that the molecular basis of therapeutic action and overall clinical efficacy of MDMA as an adjutant treatment for mental illness be further elucidated lest MDMA remain a schedule I drug, barred from use as a therapeutic. As noted by Morgan (17), drugs of abuse such as MDMA must be researched and discussed with care and thus what follows will attempt to clearly delineate between what is well-supported by evidence, what is modestly supported by evidence but in need of additional elucidation, and what is largely speculative. It is therefore the purpose of this narrative review to discuss the use of MDMA-assisted psychotherapy as a treatment for PTSD and suggest a primary mechanism of action supporting the purported efficacy of the drug. It is neither the intention of this review to provide a comprehensive accounting of the proposed mechanisms of action of MDMA as they relate to the treatment of mental illness nor dimmish the importance of the pro-social effect of the drug and the role it may play in facilitating therapeutic alliance. Rather we suggest a relationship between the current model of pathological memory encoding and inhibited memory extinction with subsequent reconsolidation in PTSD and the action of MDMA to reverse or ameliorate this defect (18, 19).

## PTSD

Post-Traumatic Stress Disorder (PTSD) is a debilitating, often chronic illness triggered by exposure to a single or a series of traumatic events and is characterized by impaired functioning in multiple functional domains (20). The World Health Organization (WHO) developed the International Classification of Functioning, Disability, and Health (ICF) to provide a common language for use when discussing disability and health which described functional “domains,” or activities and tasks where impairment is disruptive of quality of life (21, 22). A systematic review conducted by Jellestad et al. (23) found PTSD has been associated with impairment in the ICF domains of “General Tasks and Demands, Mobility, Self-Care, Domestic Life, Interpersonal Interactions and Relationships, Major Life Areas and Community, Social and Civic Life” (p. 14). The DSM III criteria for PTSD was based on dysfunctions in three domains: hypervigilance, the re-experiencing of the original trauma, and avoidant behavior of stimuli associated with the original trauma (24). In 1994, the DSM IV revised and expanded the diagnostic criteria for PTSD, which were subsequently widely criticized for reasons as disparate and contradictory as being too narrow in defining trauma to being over-diagnosed and thus too sensitive in populations recently exposed to traumatic events (25, 26). The DSM, published in 2013, again significantly revised the diagnostic criteria for PTSD. Most notably, the disorder was reclassified from an anxiety disorder to a new category dedicated to disorders associated with traumatic events. Briefly, a diagnosis of PTSD can be made when: (a) a person has been exposed to a single traumatic or to repeated traumatic events either directly or indirectly; (b) the person must experience at least one intrusive symptom such as flashbacks; (c) two or more negative mood or cognitive alterations; and (d) two or more symptoms of increased arousal such as hypervigilance (27, 28). Diagnosis can be complicated, however, because the ICD-11 defines PTSD more narrowly; in trauma survivors, it has been suggested that only 42% met both the ICD-11 and DSM-V criteria for PTSD (29). PTSD is also associated with a significant increase in physical/medical morbidity and premature mortality (30–32). Patients with a diagnosis of PTSD are at greater risk disorders of multiple organ systems including the musculoskeletal and cardiovascular system and frequently suffer from a variety of comorbid psychiatric illnesses. For patients with recurrent symptoms, PTSD increases the risk of accelerated cognitive decline (dementia) (33). Suicide risk is also thought to be elevated in this patient population, although the high rate of comorbid psychiatric diagnoses has been confounding (34, 35). One recent study, however, reported suicide rates that were 6.74 times higher for men with PTSD and remained elevated at 2.61 times the basal rate in the studied population after controlling for comorbid psychiatric illness (36).

Once diagnosed, the treatment of PTSD is complicated and often ineffective, with between 40 and 70% of patients diagnosed with the disorder suffering recurrent symptoms despite treatment (1, 37, 38). The treatment modality—pharmacological or psychotherapeutic—seems to matter little; even with large effect sizes in RCTs. First line psychotherapies like cognitive processing therapy (CPT) and prolonged exposure therapy (PET) only induce complete remission in 28–40% of patients (39). The lack of agreement between the multiple treatment guidelines produced by organizations as varied as the American Psychiatric Association (APA), the Veterans Health Administration (VA), the American Academy of Family Physicians (AAFP), and several other public and private organizations is problematic. This also creates a dilemma for providers attempting to select appropriate, evidence-based therapies for their patients (40). The VA (41) and APA guidelines, for instance, do not agree on first-line treatment for PTSD, with the APA recommending psychotherapy and pharmacotherapy (42) as equally effective options while the VA considers traumafocused therapies superior to pharmacotherapy (43). It is beyond the scope of this article to describe and compare the many modalities that have been studied for the treatment of PTSD; a summary is provided below.

## PTSD treatment

### Pharmacological

As of 2019, only two medications– the selective serotonin reuptake inhibitors (SSRI) paroxetine (Paxil^®^) and sertraline (Zoloft^®^) have full FDA approval for the treatment of PTSD (43). Although FDA approval has not been conferred, sufficient evidence from randomly controlled trials suggests that Fluoxetine (Prozac^®^) and the serotonin and norepinephrine reuptake inhibitor (SSNI) venlafaxine may also be effective in the treatment of PTSD. The atypical antipsychotic quetiapine (Seroquel^®^) may also be superior to placebo as a monotherapy (44, 45). Besides SSRIs, other classes of medications trialed for PTSD treatment include α-adrenoreceptor antagonists (prazosin), atypical antipsychotics (risperidone, olanzapine), atypical antidepressants (trazodone, nefazodone, mirtazapine), MAOIs (brofaromine, phenelzine), tricyclic antidepressants (imipramine), anticonvulsants (topiramate, valproic acid, tiagabine), β-blockers (propranolol), and antihistamines (hydroxyzine) (44–46). Recently, molecules with hallucinogenic and/or dissociative properties like ketamine, psilocybin, and MDMA have generated considerable interest as therapeutics for a variety of psychiatric illnesses including PTSD (47, 48). Failure to separate from placebo has been of significant concern in many of the drugs trialed as PTSD treatments; in one study for example, citalopram did not show benefit over placebo but qt prolongation, a known side effect, was seen in the experimental arm of the trial (43). In general, most of the medications trialed for PTSD treatment demonstrated improvements in clinician or patient rated symptom scales but did not show benefit over placebo (46). Huang et al. (46) and Hoskins et al. (45) conducted thorough meta-analyses of the efficacy of pharmacotherapy for PTSD and are excellent sources for addition information regarding the medical treatment of the disorder.

### Psychotherapies

As noted above, psychotherapy is a first-line treatment for PTSD. Broadly, psychotherapies for PTSD can be divided into two categories: Trauma-focused treatments and non-trauma-focused treatments (40). In trauma-focused therapy, the traumatic event and accompanying negative emotions are directly confronted while non-trauma-focused therapies instead target PTSD symptomatology and maladaptive coping mechanisms without addressing the underlying insult (49, 50). Although it has been suggested that trauma-focused therapy might be less well-tolerated by PTSD patients and therefore lead to increased treatment drop out, patients prematurely terminate treatment with both trauma-focused and non-trauma-focused at equal rates (51, 52). Numerous psychotherapeutic modalities have been tested for the treatment of PTSD; a recent systematic review and meta-analysis by Lewis et al. (50) included studies of 29 discreet interventions. Of the modalities reviewed, Cognitive Processing Therapy (CPT), Prolonged Exposure Therapy (PE), Cognitive Therapy (CT), and Eye Movement Desensitization Therapy (EMDT) had the most robust data supporting their efficacy with several additional modalities demonstrating promising efficacy, but requiring additional study (28, 40, 50). Analysis of long-term psychotherapeutic treatment for PTSD supports the efficacy of the same treatment modalities (49). Mindfulness-based therapies such as Mindfulness-Based Stress Reduction (MBSR) are becoming of interest as an alternative to the techniques above, particularly for patients who prefer a non-trauma-focused approach; the majority of highly effective psychotherapeutic modalities for PTSD are trauma-focused (39).

### Pharmacological-assisted psychotherapy

The use of psychedelics to enhance the clinical benefits of psychotherapy is not a new idea, with experiments in the 1950–1970's trialing “classic” psychedelics like LSD in the treatment of several psychiatric illnesses (14, 53). The scheduling of LSD, MDMA, and other psychedelics has made research more difficult, but these molecules have recently become of increased interest due to the need for more effective treatments for psychiatric illnesses like PTSD (54, 55). A simple search of PubMed using the term “psychedelics” returned 1,510 possible matches published in the past 12 months alone. Psychotherapy “enhanced” or assisted by classical psychedelics like psilocybin or non-classical hallucinogenic molecules such as Ketamine and MDMA (referred to hereafter as “psychedelic-assisted therapy” for simplicity) may be the most promising use of these medications (45, 55–57). In psychedelic-assisted psychotherapy, patients usually attend a limited number of “preparatory” psychotherapy sessions followed by administration of a fixed dose of a psychedelic in a supportive setting where therapists are present throughout the experience; often the psychedelic-enhanced session may last as long as 8–12 h and involve two or more therapists working in shifts, acting as facilitators of the session and not active directors (17). Patients then attend follow-up sessions after their psychedelic experience (58, 59). Although randomly controlled studies of psychedelic-assisted therapy have generally been small, the results suggest that the treatment modality produces significant and durable improvements in psychiatric symptomology. For example, one 2011 quasi-crossover randomly controlled trial of 20 patients diagnosed with treatment resistant PTSD treated with MDMA-assisted psychotherapy found a clinically significant decrease in Clinician-Administered PTSD Scale scores in 83% in the treatment arm vs. 25% in the placebo arm. Long-term follow up found that 14/19 patients enrolled in the trial had a durable improvement of PTSD symptoms three and half years after treatment (60, 61). Although still potentially under-powered, a meta-analysis of six stage-two clinical trials found that 53% of subjects in the active treatment groups no longer met the clinical criteria of PTSD as much as 6 years after treatment with MDMA-assisted psychotherapy as compared to 23% in controls (17, 18). Based on the pooled results of similar trials, the Food and Drug Administration (FDA) approved MDMA-assisted therapy as a break-through treatment for PTSD (6). Studies are ongoing, investigating both the overall efficacy of psychedelics in the treatment of PTSD as well as their effect on specific symptoms such as disturbed sleep and persistent nightmares (62).

## MDMA

### History and chemistry

3,4-methylenedioxymethamphetamine (MDMA) is a phenethylamine amphetamine derivative with structural similarity to classic psychedelics like mescaline (63–65). MDMA was synthesized for the first time in 1912 by chemist Arthur Kollisch and patented by Merck as a potential hemostatic agent (66). Alexander T. Shulgin “rediscovered” MDMA in 1965 after synthesizing the related compounds 3,4-methylenedioxyethylamphetamine (MDE), 3-methoxy-4,5-methylenedioxyamphetamine (MMDA), and 3,4-methylenedioxyamphetamine (MDA) in the early 1960's. MDMA can be synthesized via a variety of processes that generally result in a racemic mixture of products. More recently, the production of enantiopure MDMA has increased to explore the differential pharmacology of the (S) and (R) forms. Differential binding of the (S) and (R) isomers occurs at the 5HT_1_ and 5HT_2_ receptors but the psychopharmacological effects of the stereospecific binding of MDMA are unclear. The (S) isomer may be associated with the empathic and psychostimulant of MDMA and possibly the (R) isomer with the hallucinogenic effects of the drug (63); Lyon et al. (67, 68). Shulgin synthesized and investigated the psychoactive properties of over 200 compounds from 1960 to 1993, including MDMA and was the first to note the unique properties of MDMA intoxication. Historical reconstruction suggests that MDMA was first used as a street drug in the late 1960's, and increasingly detected in seized illicit substances by forensic laboratories throughout the 1970's (66). Like LSD before it, in the 1980's MDMA was explored as a possible pharmacotherapy for a variety of psychiatric disorders and as an adjunctive to talking therapy. Unfortunately, MDMA rebranded as “Ecstasy,” became popular as a recreational drug in the club scene of the 1980's and by 1985 had been designated as a Schedule I compound with high abuse potential without any known medical use (14). As of 2004, a reported 11 million people in the United States had experimented with MDMA at some point in their lives (69). By 2011, the number of Americans reporting experimentation with MDMA increased to 14.5 million (10).

### Physiological and psychological effects

As noted above, the effects of MDMA consumption are unique, with stimulatory, hallucinogenic, and pro-social components that defy the easy categorization and inclusion of MDMA in other classes of drugs such as the “classical” psychedelics or other amphetamine-based, psycho-stimulant molecules (13, 64). The pro-social effects of MDMA have resulted in its description as a novel drug termed an “Entactogen” or “empathogen” (14, 64, 70). The subjective effects of MDMA have been amply described in numerous studies, with sexual arousal, euphoria, relaxation, sensory enhancement, and mild visual hallucinations frequently described (10, 71, 72). Importantly, particularly in the setting of its use as an adjunctive to talking theory, is the increased sociability induced by MDMA (71). As noted by Vegting et al. (73), ecstasy comes from the Greek “ε*κστασις*” (ekstasis) which roughly translates as “standing outside of yourself” (p. 3,473). The pro-social effects of MDMA have also been well-described and include an enhanced sense of trust, improved empathy, an increased sense of closeness and inclusion, and a general increase in social confidence/perceived social competence (11, 12, 70). Kamilar-Britt and Bedi (70) provide an excellent review of lab and clinical research regarding the pro-social effects on MDMA. Physiologically, not all the effects of MDMA are reported as positive by its users, with undesirable effects such as headache, nausea, bruxism, trismus, agitation, and dry mouth, racing heart, fever, chills, and insomnia sometimes experienced (10, 12, 74). Further, long-term use of MDMA has been associated with multiple sequelae including depressed mood and anxiety; cognitive deficits in working memory, attention, and verbal processing; and a reduction in serotonin transporters (SERTs) resulting in escalating tolerance (75). Multiple studies have explored the potential neurotoxicity of MDMA in the setting of long-time use (24, 69, 76). Thus, it is important to balance the therapeutic benefits of MDMA against the negative effects of chronic use– in general; the use of MDMA to treat mental illness is limited to two or three carefully controlled administrations thus alleviating concern for the long-term consequences sometimes experienced by recreational users (14). Recently, Mitchell et al. (77) reported the results of a randomized, double blind, multi-site, placebo-controlled stage three clinical trial (*n* = 90) of MDMA for the treatment of severe PTSD. The authors concluded that MDMA as compared to placebo produced statistically significant improvement in PTSD symptom serenity as measured by the Clinician-Administered PTSD Scale for DSM-V (CAPS-5), the primary endpoint of the study (77).

### Biochemistry and pharmacology

The psychological and physiological effects of MDMA are a result of a complex set of only partially understood interactions between the drug and multiple neurotransmitter pathways (69). MDMA readily crosses the blood-brain barrier and binds to multiple targets with varying affinity, producing its effects in a dose-dependent manner (78). Like other psychostimulants, MDMA (the [+] enantiomer) binds with high affinity to monoamine transporters; the drug has the greatest affinity for the norepinephrine transporter (NET; K_M_ = 225 ± 113 nM), followed closely by the dopamine transporter (DAT; K_M_ = 444 ± 227 nM) and the serotonin transporter (SERT; K_M_ = 447 ± 197 nM) (79). These transporters clear monoamines from the synaptic cleft and move them across the neuronal membrane where they are subsequently repackaged into storage vesicles by the vesicular monoamine transporter (VMAT) for later release (80). Verrico et al. (79) noted, however, that binding site affinity and subsequent transporter blockade do not necessarily have a linear correlation, suggesting that full inhibition was inducible regardless of affinity. However, as opposed to stimulants like cocaine that only blockade monoamine transporters, MDMA is a full substrate of SERT, DAT, and NET; it not only competes with endogenous monoamine substrates, upon binding it reverses the direction of transport and induces non-exocytic/non-vesicular, transporter-mediated release of serotonin, dopamine, and norepinephrine into the synaptic cleft (76, 81, 82). Additionally, MDMA binds to the VMAT_2_ transporter and by reversing the direction of transport and reducing the pH gradient across the vesicular membrane, induces the release of monoamines into the cytosolic pool of the neuron (83). The combination of inhibition and reversal of reuptake and the suppression of vesicular sequestration of monoamines inside the neuron results in a rapid increase in neurotransmitter levels at the synaptic cleft, producing many of the acute effects of MDMA (81). MDMA is also thought to bind directly to the dopamine D1 and D2, β-adrenergic, α2-adrenergic, serotonin 5HT2A, serotonin 5HT2B, and H1-histaminergic receptors. Activity at these receptors is relatively weak however, and it is not yet clear how interaction at these receptors contributes to the effects of the drug (78, 84, 85). Recent studies suggest that MDMA may also be active at the trace-amine associated receptor (TAAR1) and the Sigma-1 receptor as well (63). Significant increase in serum levels of oxytocin, prolactin, dehydroepiandrosterone (DHEA), vasopressin, and cortisol are also seen after MDMA ingestion, but these increases may be a result of increased serotonergic activity and not a direct effect of the drug itself. This conclusion is supported by a recent study that found that oxytocin release and the pro-social behavior resulting from increased plasma levels of oxytocin was extinguished by administering a 5HT1A antagonist in a murine model (63). These acute physiological effects account for the transient euphoria, increased sociability, and hallucinations induced by MDMA, but they do not provide an immediate explanation for the efficacy of MDMA-assisted therapy as a treatment for PTSD (18).

### Fear learning and extinction

Although it is beyond the scope of this review to extensively explore the complex bi-directional interaction between emotion, physiology, and memory, decades of research support the theory that emotional and physiological state determine in part what is remembered (86–88). Importantly, emotional state at the time of memory formation may be retrieved and re-experienced when external stimuli similar to those present when the memory was encoded are encountered again (89, 90). As noted by Engen and Anderson (89), exogenously stimulated emotional states and the episodic memories that often accompany these states can be disruptive, with perturbations of equilibrium inversely correlated with an individual's ability to self-regulate their emotional state. Fear is an essentially universal response to perceived threats that allows animals to adopt an appropriate defensive posture preparatory to fighting or fleeing without the time lag associated with conscious cognitive processing. Fear learning, or the association of an unconditioned stimulus (US) with a conditioned stimulus (CS), is an adaptative process that allows organisms—including humans—to reflexively respond to an initially novel environmental threat should exogenous cues similar to those present at the original encounter be encountered again (90, 91). These learned responses can persist for a lifetime (92).

Fear learning is thought to be regulated by a neural network connecting the amygdala, the medial pre-frontal cortex (mPFC), and the hippocampus (93, 94). The amygdala, a part of the limbic system, is a complex collection of nuclei that participates in memory processing, emotional expression, and the management of sensory input. It is located superior to the brainstem, and is an evolutionarily older structure (95). The pre-frontal cortex is a “younger” part of the brain, located deep to the frontal bone in the anterior-most part of the brain, and is thought to be the part of the brain from which cognition, decision-making, and other complex, higher-function processes arise (96). The hippocampi are two mirrored structures consisting of three parts: the cornu ammonis, dentate gyrus, and subiculum (97). The role of these structures in fear memory learning has been elucidated primarily using disruptive pharmaceuticals and surgical lesioning techniques to demonstrate the inhibition of the development of a persistent fear response when any of these pathways are interrupted (90). Much simplified, the fear learning process integrates sensory information coding for both the US and CS, sent to the lateral nucleus (LA) of the amygdala both directly from the primary sensory cortices and thalamus and indirectly through the cortico-amygdala pathway. The overlapping US and CS signals strengthen neuronal connections in these pathways, potentiating the fear-learning process; neuronal plasticity at the molecular level is thought to account for this effect (98–102). The signals arising in the LA are then relayed to the basolateral (BLA) and central nucleus of the amygdala, the later triggering the physiological fear response through projections to the hypothalamus and other targets in the brainstem. The BLA connects to the ventral hippocampus and plays a critical role in attaching context-related cueing to the fear response (90, 91, 103). The medial pre-frontal cortex also connects directly to the BLA, modulating behavioral responses to fear and plays a critical role in the extinction of fear memory learning (90). In molecular terms, multiple pathways and neuromodulators are implicated in the process, including brain-derived neurotrophic factor (BDNF) and its associated receptor tropomyosin-related kinase B (Trk-B), multiple gene transcription factors, and a diverse set of metabotropic and ionotropic receptors (100, 104). Cahil and Milton (104) have provided a comprehensive summary of these molecular mechanisms.

As noted above, although fear memory learning is often adaptive, the conditioned response to benign stimuli can be extremely disruptive; once a threat is no longer present, it is maladaptive to retain an associated fear response (101, 105). Thus, it is also adaptive that fear memory learning can be “unlearned,” a process termed extinction (106, 107). Fear extinction has been defined as “…a lessening of conditioned fear responses following extinction training, during which subjects are exposed to repetitive presentations of conditioned stimuli (CS) alone” [(110), p. 1]. Although the process is not entirely understood, fear extinction has been thought to involve either the uncoupling of the response from the fear memory, the selective erasure of the fear memory, or perhaps both (92). Memory retrieval is an active process, in which the recalled memory is destabilized transiently, restabilized in a process called reconsolidation, then returned to quiescent, long-term storage state (108). Recently, Kida (108, 109) has presented data that suggested that fear memory extinction is better characterized as a learning event in which an inhibitory memory is encoded by the same process, in the same brain structures, as the original fear memory. Supporting this hypothesis, a study of the role of dopamine in fear memory extinction concluded that dopaminergic neurons projecting from the BLA and a set of contiguous structures (intercalated cell masses), combined with glutaminergic neurons in the mPFC, specifically the infra-limbic subregion, were all highly active during fear memory extinction. Selectively ablating these neurons suppressed the extinction of fear memories in a murine model (105, 110, 111). Further, multiple studies have suggested that synaptic plasticity in the mPFC, the hippocampus, and the BLA is as essential in extinguishing fear memories as it is in forming them (99–101).

### Pathological fear learning and extinction in PTSD

The pathophysiology of PTSD, while well-studied, remains incompletely understood (112). As noted by Krystal et al. (113), defining PTSD solely as a deficit in fear memory learning extinction is likely too narrow. Nevertheless, the fear memory learning pathways discussed above demonstrate significant, apparently pathological changes leading to dysfunctional fear memory learning defects in patients suffering from the illness (93, 114, 115). It has been suggested that the intrusive symptoms of PTSD—disturbing nightmares, upsetting memories, and uncontrollable flashbacks—have been correlated with impairment of fear memory extinction and may also underly additional symptoms such as pervasive feelings of guilt and shame, anxiety, and predilections for uncontrollable anger and substance abuse (38, 109, 115–118). Several review articles have explored the central role of impaired fear memory learning extinction in the pathogenesis of PTSD (93, 113, 116, 118). These studies found moderately consistent evidence that there are pathological changes in the amygdala, hippocampus, and pre-frontal cortex at the functional, structural, and molecular levels.

Functionally, PTSD patients have displayed increased amygdala activation as assessed by fMRI during fear conditioning as opposed to healthy, non-trauma exposed controls (93, 115, 117–119). Hyperactivity in the BLA in particular may be associated with increased fear response to environmental conditions and a corresponding tendency to generalize threat responses to benign stimuli (93, 115, 118, 120, 121). It has also been suggested that in PTSD patients, the amygdala shows increased responsiveness to negative stimuli in general (120). Several studies have also found increased activity during fear conditioning in the dorsal anterior cingulate cortex (dACC) and reduced responsiveness in the ventral anterior cingulate cortex during fear memory extinction (114, 115, 122). Reduced activity in the ventral-medial pre-frontal cortex (vmPFC) has been reported in several fMRI studies, particularly during extinction training recall (93, 115, 117). It has been suggested that in healthy patients, the vmPFC suppresses the amygdala's response to negative stimuli, and regulates fear memory extinction and extinction recall (93, 114, 118, 123). Hippocampal hypoactivity has also been found in PTSD patients, possibly indicating impairment of context-dependent memory encoding during both fear memory learning and extinction. Further, like the vmPFC, the hippocampus is thought to regulate activity in the amygdala and thus hippocampal dysfunction may also play a role in amygdala hyperactivity (115–117, 124). Additionally, functional impairment in *connectivity* between these brain regions and others may significantly contribute to the symptomology of PTSD (101, 113, 122, 123). It is important to note that there has been variability in fMRI results, measuring brain activity in PTSD patients. The divergence in findings may be due to atypical vs. typical PTSD presentations, genetic variations resulting in baseline differences between patients, the stimuli used to elicit fear responses in the patients, and variances in experimental setting (93). Likewise, structural analysis of the brain regions involved in fear memory learning produced mixed and sometimes conflicting results (93, 121, 125). Regardless of specific pathology, functional dysregulation of fear memory learning is consistent in patients with PTSD.

In PTSD, pathological changes at the neuronal/molecular level are likewise complex and have been extensively explored in murine models (112, 118, 126). Further, alterations in gene expression, receptor populations, neurotrophic molecule levels, neurotransmitter levels, and neuro-remodeling via changes in dendritic spine density have been found to be exposure phase-dependent; upregulations seen shortly after exposure to trauma may be transient and later resolve (109). Additionally, the population of receptors, ligands, and intracellular signaling pathways varies depending on the mechanism in question. For example, in the amygdala, the mTOR signaling pathway, the β-adrenergic receptor, the glucocorticoid receptor, β-arrestin-2, and altered glutaminergic signaling have been associated with hyperresponsiveness to negative stimuli and other PTSD-associated symptoms. An entirely different list including dopamine receptors D1 and D5 have been associated with the memory destabilization necessary for extinction and reconsolidation (109, 119). A loss of synaptic plasticity secondary to chronic inflammation, reductions in the absolute number of α-amino-3-hydroxy-5-methyl-4-isoxazloepropionic acid receptors (AMPARs) and N-methyl-D-aspartate receptors (NMDARs), reduced activity of neurotrophic molecules, increased excitotoxicity, and reduced dendritic spine density and arborization has been associated with a number of pathologies in PTSD (101, 109, 112, 118).

Brain-derived neurotrophic factor (BDNF) and its associated receptor, tropomyosin receptor kinase B (TrkB), have come under increasing scrutiny for their role in several psychiatric disorders including PTSD (100, 104, 107, 113, 127–131). The synthesis of BDNF precursor pro-BDNF occurs in microglia, astrocytes, and glutaminergic neurons which is secreted along with the mature form of the neurotrophin, both of which are active (127). BDNF signaling is associated with multiple aspects of neuroplasticity including the upregulation and phosphorylation of NMDARs and increases in the complexity, size, and number of dendritic spines at the synapse and is necessary for long-term potentiation and memory consolidation (132, 133). In PTSD, BDNF in the hippocampus is essential for fear memory extinction, and it has been suggested that a deficit of BDNF may underly hippocampal synaptic loss and the subsequent inhibition of the extinction process (113, 127, 129). The impact of BDNF activity in the amygdala (and the dACC) is less clear in PTSD and is locus dependent. BDNF in the BLA is active during the formation of a fear memory and thus an excess could be associated with worsening of PTSD symptomatology (128). Conversely, several studies have demonstrated that much like in the hippocampus, BDNF availability in the amygdala is critical for fear memory extinction and when blocked, disrupts extinction (134). Likewise, decreased BDNF activity in the vmPFC has been associated with defective fear memory extinction– in murine model, mice with BDNF Val66Met mutation that downregulates the activity-dependent secretion of the neurotrophin had reduced activity in the vmPFC during dear extinction processing (128). Additional mouse studies have demonstrated that reduced expression of BDNF has been correlated with dendritic arbor deficiencies in the hippocampus and vmPFC, inducing PTSD-associated behavioral patterns (135). Interestingly, there is conflicting data as to whether serum and/or plasma levels of BNDF are increased or decreased in PTSD, with several studies supporting both positions (128, 130). Regardless, the evidence strongly supports the assertion that aberrant BDNF signaling is a part of the pathology of PTSD; the neuroplasticity required to form new fear memory associations requires the presence of BDNF as does the neuroplasticity required for extinction learning and memory reconsolidation. In essence, the reduction of BDNF availability after trauma-induced fear memory learning “locks” the conditioned response in place, preventing extinction and memory reconsolidation.

### Traumatic memory extinction and reconsolidation mediated by MDMA

As discussed above, the efficacy of MDMA as an adjunctive enhancer of psychotherapy in the treatment of PTSD is mediated by multiple mechanisms, some of which likely remain unexplored. Many mechanisms have been postulated. Smith et al. (136), for instance suggest that MDMA bilaterally suppresses amygdala activation and thus dampens negative emotions while simultaneously enhancing responsiveness to positive emotion thus facilitating psychotherapy. Wagner et al. (57) similarly support the idea that immediate and long-term potentiation of (interpersonal) openness and repressed neuroticism mediated by serotonin and oxytocin release may explain MDMA's effect in improving PTSD symptoms. Vermetten and Yehuda (137) likewise suggest that the altered state of consciousness induced by MDMA “can facilitate a deeper psychotherapeutic process” (p. 231). The pro-social effects of MDMA are well-known and likely contribute, perhaps significantly, to efficacy of MDMA-assisted psychotherapy (138, 139). In this model, the primary effect of MDMA in the treatment of PTSD is to enhance the strength of the therapeutic alliance between patient and provider (139). This effect alone, however, does not comfortably account for the effect of MDMA as a therapeutic in light of the functional, structural, and molecular neuronal deficits found in PTSD, particularly in the fear memory leaning and extinction pathways.

However, like other psychedelics such as psilocybin, MDMA has been demonstrated to induce increases in neural plasticity (63, 140–144)). Ly et al. (145) and Olsen (146) suggested that the term “psychoplastogen” might be an appropriate label for molecules such as MDMA, psilocybin, and ketamine because of their profound neurogenic effects. The authors further noted that the neuroplastic effects of the “psychoplastogens” like MDMA are BDNF-dependent; even some studies discussing the addictive and neurotoxic effects of MDMA have noted the alterations in BDNF expression in many regions of the brain after administration of the drug in murine models (140, 145–148). Despite some evidence that serotonergic signaling and transport plays a vital if not necessary role in MDMA's ability to enhance (or at least dis-inhibit) fear memory extinction, BDNF signaling was the necessary component for the effect to occur (107, 140, 145). Multiple studies found increased BDNF protein levels or increased BDNF mRNA transcripts in regions of the brain critical to fear learning such as the hippocampus and the vmPFC after MDMA treatment. Dunlap et al. (63) for instance noted that significantly increased levels of BDNF mRNA transcripts were found in the prefrontal cortex of rats after MDMA administration, with increases of BDNF's target receptor TrkB also noted 24 h after drug treatment. Although Martinez-Turrillas (149) conversely found decreased levels of BDNF in the hippocampus in their study, other authors have found the opposite and report increased Hippocampal BDNF in the dentate gyrus and CA3 specifically (150). In these areas of the brain where it has been suggested that the pathology of PTSD induces neuronal loss and degraded synaptic connections, upregulation of BDNF mediated by MDMA has resulted in increased synaptic connectivity and growth in dendritic spine density and number (145, 150). Further supporting the importance of BDNF in the neuroplastic and therefore therapeutic effects of MDMA, the inhibition of BDNF, its receptor TrkB, or TrkB's downstream signaling molecule mTOR resulted in complete inhibition of psychedelic-induced neuronal plasticity (107, 140, 143, 145, 146). Thus, MDMA, by upregulating or at least restoring normal levels of BDNF in the amygdala, hippocampus, and vmPFC, may “unlock” the trauma-induced fear memory association and allows for extinction learning and subsequent reconsolidation of the memory. This mechanism, in addition to the pro-social effects that allow for a deeper therapeutic alliance between patient and provider may account for a significant part of the efficacy of MDMA in the treatment of PTSD.

## Discussion and conclusion

MDMA-assisted psychotherapy, though approved as a breakthrough treatment by the FDA, will require significant additional research before it can be accepted as a first-line option for refractory PTSD (6, 17). Nevertheless, over the past two decades, enough evidence has accumulated to postulate empirically supported rationale for the reported efficacy of the adjunctive use of MDMA. While the pro-social effects of MDMA are profound and likely substantially improve therapeutic alliance, without an additional mechanism of action working at the molecular level, it is difficult to account for the large effect size reported for MDMA-assisted psychotherapy. BDNF-facilitated neuroplasticity aligns a key pathogenic mechanism in PTSD (dysfunction in the fear memory pathways) with a known drug effect for MDMA and other psychedelics. As is often the case, the evidence here to date only demonstrates correlation and not causation and thus it is not possible to state categorically that BDNF and subsequent increases in neuronal connectivity and dendritic spine density are the motive force behind the positive effects of the treatment. Further, the direct effects of MDMA on various receptors and the massive increase in neurotransmitter availability at the synapse has not been addressed here and cannot be discounted as another explanatory mechanism for treatment efficacy (151). The difficulty in correlating molecular studies in murine models with human behavior is likewise a problem as it often is in the treatment of psychiatric illness. These limitations aside, it seems likely that BDNF modulation by MDMA accounts for at least a part of the theoretic benefits associated with MDMA-assisted psychotherapy for the treatment of PTSD. Future studies will continue to sharpen the model presented here, or suggest an alternative model; either way, MDMA-assisted therapy provides another option for patients suffering from PTSD that have failed multiple treatment regimens.

## Author contributions

All authors listed have made a substantial, direct, and intellectual contribution to the work and approved it for publication.

## Conflict of interest

The authors declare that the research was conducted in the absence of any commercial or financial relationships that could be construed as a potential conflict of interest.

## Publisher's note

All claims expressed in this article are solely those of the authors and do not necessarily represent those of their affiliated organizations, or those of the publisher, the editors and the reviewers. Any product that may be evaluated in this article, or claim that may be made by its manufacturer, is not guaranteed or endorsed by the publisher.
